# Dysfunction of the RAR/RXR signaling pathway in the forebrain impairs hippocampal memory and synaptic plasticity

**DOI:** 10.1186/1756-6606-5-8

**Published:** 2012-02-08

**Authors:** Masanori Nomoto, Yohei Takeda, Shusaku Uchida, Koji Mitsuda, Hatsune Enomoto, Kaori Saito, Tesu Choi, Ayako M Watabe, Shizuka Kobayashi, Shoichi Masushige, Toshiya Manabe, Satoshi Kida

**Affiliations:** 1 Department of Bioscience, Faculty of Applied Bioscience, Tokyo University of Agriculture, Tokyo 156-8502, Japan; 2 Core Research for Evolutional Science and Technology, Japan Science and Technology Agency, Saitama 332-0012, Japan; 3 Division of Neuronal Network, Department of Basic Medical Sciences, Institute of Medical Science, University of Tokyo, Tokyo 108-8639, Japan; 4 Laboratory of Neurophysiology, Department of Neuroscience, Jikei University School of Medicine, Minato-ku, Tokyo 105-8461, Japan; Precursory Research for Embryonic Science and Technology, Japan Science and Technology Agency, Kawaguchi, Saitama 332-0012, Japan

## Abstract

**Background:**

Retinoid signaling pathways mediated by retinoic acid receptor (RAR)/retinoid × receptor (RXR)-mediated transcription play critical roles in hippocampal synaptic plasticity. Furthermore, recent studies have shown that treatment with retinoic acid alleviates age-related deficits in hippocampal long-term potentiation (LTP) and memory performance and, furthermore, memory deficits in a transgenic mouse model of Alzheimer's disease. However, the roles of the RAR/RXR signaling pathway in learning and memory at the behavioral level have still not been well characterized in the adult brain. We here show essential roles for RAR/RXR in hippocampus-dependent learning and memory. In the current study, we generated transgenic mice in which the expression of dominant-negative RAR (dnRAR) could be induced in the mature brain using a tetracycline-dependent transcription factor and examined the effects of RAR/RXR loss.

**Results:**

The expression of dnRAR in the forebrain down-regulated the expression of RARβ, a target gene of RAR/RXR, indicating that dnRAR mice exhibit dysfunction of the RAR/RXR signaling pathway. Similar with previous findings, dnRAR mice displayed impaired LTP and AMPA-mediated synaptic transmission in the hippocampus. More importantly, these mutant mice displayed impaired hippocampus-dependent social recognition and spatial memory. However, these deficits of LTP and memory performance were rescued by stronger conditioning stimulation and spaced training, respectively. Finally, we found that pharmacological blockade of RARα in the hippocampus impairs social recognition memory.

**Conclusions:**

From these observations, we concluded that the RAR/RXR signaling pathway greatly contributes to learning and memory, and LTP in the hippocampus in the adult brain.

## Background

Retinoic acids (RAs) are biologically active metabolites of vitamin A, an essential nutrient factor [1-3]. Vitamin A-RA signaling pathways play essential roles in a wide range of biological functions such as reproduction, growth, differentiation, development, vision, and homeostasis of various tissues, including the brain [2,4].

*All-trans*-RA and *9-cis*-isomers of RA bind to their nuclear receptor, i.e., RA receptors (RARα, β, and γ) and retinoid × receptors (RXRα, β, and γ), which function as ligand-inducible transcription factors [4,5]. RA binding to RAR and RXR, respectively, forms a heterodimer of RAR/RXR or a homodimer of RXR/RXR and regulates the transcription of target genes by binding to retinoic acid responsive elements in their promoter regions, thereby regulating various biological phenomena [4,6].

RAR and RXR, especially RARα are highly expressed in a wide range of central nervous system tissues including the mature brain [7,8]. Moreover, there is growing evidence that vitamin A-RA signaling pathways have an impact on higher brain function; furthermore, an impairment of these signaling pathways is implicated in the etiology of Alzheimer's disease and psychiatric disorders such as schizophrenia [9-15]. Indeed, recent studies using mice have shown that age-related memory deficits are associated with the hypo-function of vitamin A-RA signaling pathways and these deficits are restored by treatment with RA or supplementation of vitamin A [16,17].

Importantly, deletion of the RARβ or RARβ/RXRγ genes leads to deficits in hippocampal synaptic plasticity, e.g., long-term potentiation (LTP) and long-term depression (LTD) [18]. Similarly, vitamin A-deficient mice display impaired hippocampal LTP that is rescued by treatment with RA [19,20]. These findings strongly suggest that the RAR/RXR signaling pathway plays critical roles in hippocampal LTP and LTD. However, the roles of the RAR/RXR signaling pathway in learning and memory have not been well characterized in the mature brain, especially in the hippocampus. Indeed, previous genetic studies have shown that mice lacking the RARβ or RARβ/RXRγ genes displayed deficits in motor coordination that made it difficult to estimate their learning and memory abilities [21]. However, these studies suggested that loss-of-function of the RAR/RXR signaling pathway impairs spatial learning and memory [18]. Additionally, these studies did not exclude the possibility that genetic depletion of the RAR/RXR genes leads to some developmental changes in the brain.

In the current study, we tried to understand the roles of the RAR/RXR signaling pathway in hippocampus-dependent learning and memory and in hippocampal synaptic plasticity. To do this, we generated conditional mutant mice in which the expression of a dominant-negative mutant of RARα could be induced in the forebrain using a tetracycline-dependent transcription factor, and performed electrophysiological experiments and hippocampus-dependent memory tasks using these transgenic mice. Furthermore, we examined the effects of pharmacological inhibition of RARα in the hippocampus on memory performance.

## Results

**Figure 1:**
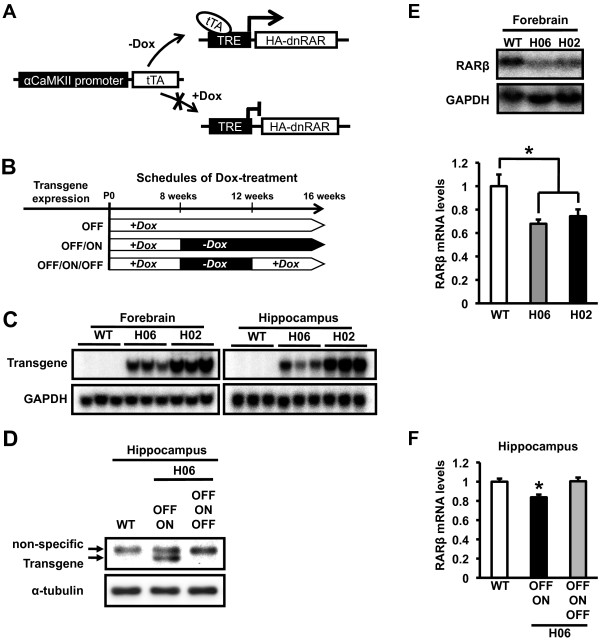
**dnRAR mice displayed Dox-dependent expression of dnRAR and down-regulation of RARβ expression in the forebrain**. (A) Schematic representation of Dox-dependent regulation of dnRAR in the forebrain. (B) Experimental design. Schedule for the treatment of dnRAR mice with Dox. The mice were treated with Dox throughout their lifetime (OFF) or until they were 8 weeks old (OFF - transgene ON; OFF/ON). OFF/ON-dnRAR mice were treated again with Dox for 4 weeks following the withdrawal of Dox for 4 weeks (OFF/ON/OFF). (C) Northern blot analysis of dnRAR mRNA in the forebrain and hippocampus of dnRAR H06 and H02 mice and WT littermates (WT). The upper and lower panels shows the expression of dnRAR mRNA (2.8 kbp) and GAPDH mRNA (1.3 kbp) as an internal control, respectively. (D) Western blot analysis of dnRAR protein in the hippocampus of dnRAR H06 mice and WT littermates. The upper panel shows the expression of a 50-kDa protein corresponding to dnRAR. The upper arrow indicates non-specific binding and the lower arrow indicates dnRAR protein. The lower panel shows the expression of α-tubulin as an internal control. (E) Northern blot analysis of RARβ mRNA in the forebrain of dnRAR H06 and H02 mice and WT littermates. The lower panel indicates the quantification of RARβ mRNA levels in the forebrain of dnRAR H06 and H02 mice and WT littermates (WT, n = 8; H06, n = 8; H02, n = 8). The levels of RARβ mRNA were normalized according to the GAPDH signal. *p < 0.05, compared with WT littermates. (F) qRT-PCR analysis of RARβ mRNA in the hippocampus of dnRAR H06 and H02 mice and WT littermates. Quantification of RARβ mRNA levels in the hippocampus of OFF/ON-dnRAR H06 mice, OFF/ON/OFF-dnRAR H06 mice, and WT littermates (WT, n = 29; OFF/ON, n = 24; OFF/ON/OFF, n = 16). The levels of RARβ mRNA were normalized according to the levels of GAPDH mRNA. *p < 0.05, compared with the other groups. Error bars indicate SEM.

RARs, especially RARα, are abundantly expressed in the forebrain, including the hippocampus [7,8]. To understand the roles of the RAR/RXR signaling pathway in learning and memory and synaptic plasticity, we examined the effects of impaired RAR/RXR function in the forebrain. To do this, we generated mutant mice in which a dominant-negative mutant of RARα (dnRAR) was expressed specifically in the forebrain using a tetracycline system [22-25]. This mutant protein, lacking the C-terminus (amino acid (aa) 403-462) of RARα (aa 1-462), forms a heterodimer with RXR, but is unable to induce transcriptional activation [26]. In these mutant mice, a tetracycline-dependent transcriptional activator (tTA) expressed in the forebrain activates the expression of dnRAR specifically in this brain region in the absence of tetracycline, whereas the expression of dnRAR is suppressed when the mice are administrated doxycycline (Dox), a derivative of tetracycline, in their drinking water (Figure 1A).

### Generation of dnRAR mice

We first generated two lines (H02 and H06) of mutant mice that express dnRAR fused with an HA-tag at the N-terminus under the control of a tetracycline-responsive element (TRE)-dependent promoter (TRE-dnRAR mice) [27]. These mutant mice were crossed with transgenic mice that express tTA in the forebrain under the control of the αCaMKII promoter, which displays strong activity in the forebrain including the hippocampus, cortex, and amygdala (CaMKII-tTA mouse) [24], generating 2 lines of CaMKII-tTA/TRE-dnRAR double transgenic mice (dnRAR H02 and H06 mice).

To decrease the effects of dnRAR expression on the development of the forebrain, transgenic mice and wild-type (WT) littermates were treated with Dox until they were 8 weeks old and they were then housed without Dox-treatment to induce the expression of dnRAR (transgene OFF - transgene ON; OFF/ON; Figure 1B). For the control groups, dnRAR H06 mice were treated with Dox throughout their lifetime (transgene OFF; OFF-dnRAR mice) and OFF/ON-dnRAR H06 mice were treated again with Dox for 4 weeks following withdrawal of Dox for 4 weeks (transgene OFF-transgene ON-transgene OFF; OFF/ON/OFF-dnRAR mice; Figure 1B).

We next performed expression analysis of dnRAR in the forebrain and hippocampus of dnRAR H02 and H06 mice. Northern blot analysis using a specific probe for the 3'-untranslated region (UTR) of dnRAR mRNA revealed that dnRAR mRNA was expressed in the forebrain and hippocampus of the 2 OFF/ON double transgenic lines (Figure 1C). Importantly, dnRAR H02 mice displayed higher levels of dnRAR mRNA expression than the dnRAR H06 mice. Consistently, Western blotting using an anti-HA antibody showed the expression of dnRAR in the hippocampus of OFF/ON-dnRAR H06 mice (Figure 1D). Importantly, the expression of dnRAR was not detectable in the hippocampus of OFF/ON/OFF-dnRAR H06 mice. These observations indicated that the dnRAR mice express dnRAR in the forebrain, including the hippocampus, in a Dox-dependent manner.

We finally examined the effects of dnRAR expression on RAR/RXR-dependent gene expression in the forebrain. To do this, we analyzed the expression levels of RARβ, a target gene of RAR/RXR [1,28], in the forebrain and hippocampus using Northern blotting and quantitative RT-PCR (qRT-PCR) (Figure 1E, F, respectively). One-way analysis of variance (ANOVA) of the Northern blotting results revealed a significant effect of genotype on RARβ expression (F _(2,21) _= 5.953, p < 0.05; Figure 1E). The *post hoc *Newman-Keuls test revealed that the expression levels of RARβ mRNA in the forebrain of OFF/ON-dnRAR H02 and H06 mice were significantly lower than in WT mice (p < 0.05). Additionally, dnRAR-H06 mice displayed slightly lower expression levels of RARβ mRNA than dnRAR-H02 mice, although this difference was not statistically significant. Similarly, qRT-PCR showed that OFF/ON-dnRAR H06 mice displayed significantly lower expression levels of RARβ mRNA in the hippocampus than WT mice (p < 0.05; Figure 1F). Importantly, this decreased expression of RARβ mRNA in the hippocampus was rescued when these OFF/ON mice were treated again with Dox for 4 weeks (OFF/ON/OFF-dnRAR mice; OFF/ON/OFF vs. OFF/ON, p < 0.05; OFF/ON/OFF vs. WT, p > 0.05), indicating that the down-regulation of RARβ mRNA is dependent on the expression of dnRAR. Collectively, these observations indicated that the expression of dnRAR decreases RARβ mRNA levels in the forebrain, including the hippocampus, suggesting that dnRAR expression leads to dysfunction of the RAR/RXR signaling pathway in these brain regions. Our results indicate that the expression levels of dnRAR in the OFF/ON-dnRAR H06 mice are sufficient to downregulate RAR/RXR-target gene expression; therefore, the subsequent electrophysiological and behavioral analyses were performed using the dnRAR-H06 transgenic line.

### Basal synaptic transmission and LTP in the hippocampus of dnRAR mice

Previous studies have shown that genetic deletion of one or two RAR/RXR subtypes impaired LTP in the CA1 region of the hippocampus [18]. To examine the effects of the impaired RAR/RXR signaling pathway on synaptic plasticity, we recorded extracellular field potentials in the CA1 region of hippocampal slices from OFF/ON-dnRAR H06 mice and WT littermates.

**Figure 2:**
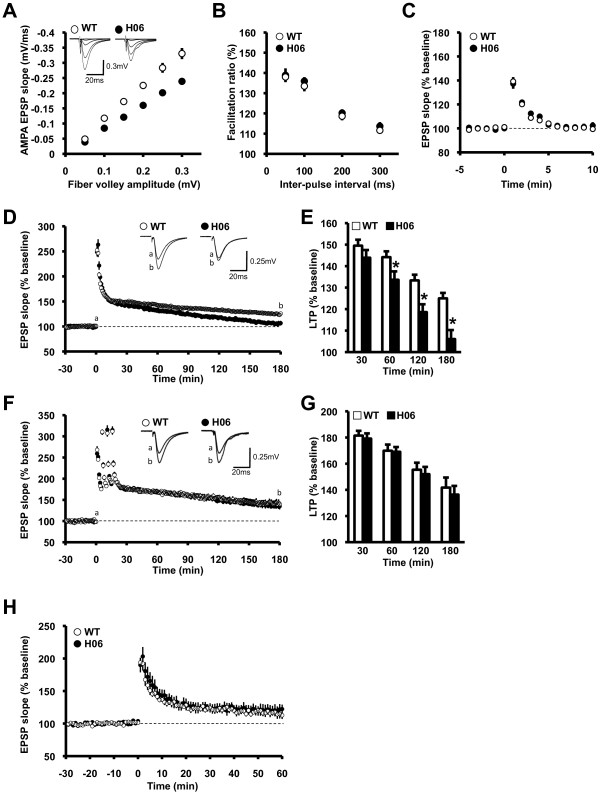
**Basal synaptic transmission and LTP in the hippocampus of dnRAR mice**. (A) The input-output relationships of AMPA receptor-mediated EPSP in WT (n = 9) and dnRAR H06 (n = 8) mice. The sample traces in the inset represent the responses evoked with the five different stimulus intensities and the stimulus artifacts were truncated. The data were first sorted by the amplitude range of the fiber volleys, and then the EPSP slopes were averaged within each range. (B) PPF induced by stimulating afferent fibers twice at intervals of 50, 100, 200, and 300 ms in WT (n = 9) and dnRAR H06 (n = 8) mice. (C) PTP induced by high-frequency stimulation (one 100 Hz, 1 s train) in the presence of D-APV (50 μM) in WT (139.2 ± 3.5% of baseline; n = 12) and dnRAR H06 (137.2 ± 4.1% of baseline; n = 10) mice. (D) LTP induced by single conditioning stimulation (one 100 Hz, 1 s train) in WT (n = 30) and dnRAR H06 (n = 16) mice. The initial EPSP slopes were measured, and the values were normalized in each experiment to the averaged slope value measured during the control period (-30 to 0 min). Conditioning stimulation was applied at 0 min. The sample traces in the inset represent the EPSPs (average of 10 consecutive responses) of WT and H06 mice recorded at the times indicated by the letters. The stimulus artifacts were truncated. (E) Summary of LTP induced by single conditioning stimulation in WT and dnRAR H06 mice (21-30 min: WT, 149.5 ± 2.0%; H06, 143.9 ± 3.5%; 51-60 min: WT, 144.1 ± 2.4%; H06, 133.6 ± 3.8%; 111-120 min: WT, 133.3 ± 2.9%; H06, 118.6 ± 3.6%; 171-180 min: WT, 125.0 ± 3.3%; H06, 106.0 ± 4.2% of baseline) (*t *test, *p < 0.05). (F) LTP induced by strong conditioning stimulation (four 100 Hz, 1 s trains at 5 min intervals) in WT (n = 6) and dnRAR H06 (n = 9) mice. The initial EPSP slopes were measured, and the values were normalized in each experiment to the averaged slope value measured during the control period (-30 to 0 min). Conditioning stimulation was applied at 0 min. The sample traces in the inset represent the EPSPs (average of 10 consecutive responses) of WT and H06 mice recorded at the times indicated by the letters. The stimulus artifacts were truncated. (G) Summary of normalized LTP induced by strong conditioning stimulation in WT and dnRAR H06 mice (21-30 min: WT, 181.4 ± 3.6%; H06, 179.2 ± 3.9%; 51-60 min: WT, 169.9 ± 4.6%; H06, 169.0 ± 3.6%; 111-120 min: WT, 155.3 ± 5.3%; H06, 151.9 ± 5.5%; 171-180 min: WT, 141.7 ± 7.7%; H06, 136.3 ± 6.6% of baseline). (H) STP induced by short conditioning stimulation (one 100 Hz, 100 ms train) in WT (116.2 ± 5.1% of baseline; n = 5) and dnRAR H06 (120.6 ± 8.1% of baseline; n = 5) mice. The initial EPSP slopes were measured, and the values were normalized in each experiment to the averaged slope value measured during the control period (-30 to 0 min). Conditioning stimulation was applied at 0 min. Error bars indicate SEM.

We investigated basal synaptic transmission in dnRAR H06 mice. We first analyzed the input-output relationships of AMPA receptor-mediated excitatory postsynaptic potentials (EPSPs) evoked by various stimulus intensities (Figure 2A). We applied a low concentration of CNQX (1 μM) to partially block the AMPA receptors because the fiber volleys were usually much smaller than the EPSPs [29,30]. dnRAR H06 mice showed significantly small AMPA synaptic responses at each presynaptic fiber volley amplitude (PSFV) compared with WT mice (p < 0.05 at each PSFV). These results suggest that AMPA receptor-mediated synaptic responses were decreased in dnRAR H06 mice.

We next analyzed paired-pulse facilitation (PPF), a presynaptic form of short-term synaptic plasticity, in dnRAR H06 mice (Figure 2B). PPF was induced by a pair of afferent fiber stimulations at short intervals (50, 100, 200, or 300 ms). dnRAR H06 mice showed comparable PPF at each inter-pulse interval compared with WT mice, suggesting that the probability of release of the neurotransmitter glutamate from the presynaptic terminal is normal in dnRAR H06 mice (each interval, p > 0.05). We also analyzed post-tetanic potentiation (PTP), which is a presynaptic phenomenon (Figure 2C) [31]. PTP was induced by conditioning stimulation (100 Hz, 1 s) in the presence of D-APV (50 μM), an NMDA receptor antagonist. The magnitude of PTP following stimulation was comparable between dnRAR and WT mice, suggesting that short-term plasticity induced by conditioning stimulation was normal in dnRAR mice (p > 0.05 at each time point).

Next, we analyzed LTP in dnRAR H06 mice. LTP was induced using a single high-frequency stimulation (one 100 Hz, 1 s train; Figure 2D and 2E). It is important to note that the stimulus strength was adjusted to obtain similar initial EPSP slope values (0.10-0.15 mV/ms) in each experiment because dnRAR H06 mice displayed impaired AMPA receptor-mediated input-output relationships compared with WT mice (Figure 2A). WT mice displayed significant synaptic potentiation following high-frequency stimulation compared with their baseline responses (p < 0.05; Figure 2D), indicating that single conditioning stimulation was sufficient to induce LTP in the hippocampal slices from WT mice. Conversely, dnRAR H06 mice also displayed significant LTP compared with their baseline responses (p < 0.05). However, the magnitude of LTP in these mutant mice was significantly lower at 51-60, 111-120, and 171-180 min following the induction of LTP compared with WT littermates (p < 0.05; Figure 2E), although the magnitude was comparable at 21-30 min between dnRAR H06 and WT mice (p > 0.05), suggesting that dnRAR H06 mice failed to maintain LTP even though LTP was induced normally. These observations indicated that dnRAR H06 mice displayed impaired LTP in the CA1 region of the hippocampus.

We examined whether stronger conditioning stimulation rescues the impaired LTP observed in the dnRAR H06 mice. To do this, LTP was induced by four high-frequency stimulations (four 100 Hz, 1 s trains at 5 min intervals; Figure 2F and 2G). WT and dnRAR H06 mice displayed significant LTP compared with their baseline responses (p < 0.05). In contrast to the results from single conditioning stimulation, dnRAR H06 mice displayed a comparable magnitude of LTP at each time point compared with WT mice. These observations suggest that strong conditioning stimulation rescued the impaired LTP observed in dnRAR H06 mice. However, we could not exclude the possibility that strong conditioning stimulation led to saturated levels of LTP, thereby masking differences in the magnitude of LTP between WT and dnRAR H06 mice.

We finally examined short-term potentiation (STP; Figure 2H). STP was induced by using shorter conditioning stimulation (100 Hz for 100 ms) than that used to induce LTP (100 Hz for 1 s). The magnitude of STP after induction was comparable between dnRAR H06 mice and WT littermates (p > 0.05). These observations suggest that dnRAR H06 mice displayed normal STP.

Taken together, our observations confirmed previous observations that dysfunction of the RAR/RXR signaling pathway results in impaired hippocampal LTP and extended these findings by demonstrating that this dysfunction also leads to an impaired AMPA receptor-mediated synaptic response. However, we could not exclude the possibility that impaired AMPA receptor-mediated EPSPs observed in dnRAR H06 mice contributed to the failure of LTP maintenance.

### Impaired social memory in dnRAR mice and its rescue by stronger training

**Figure 3:**
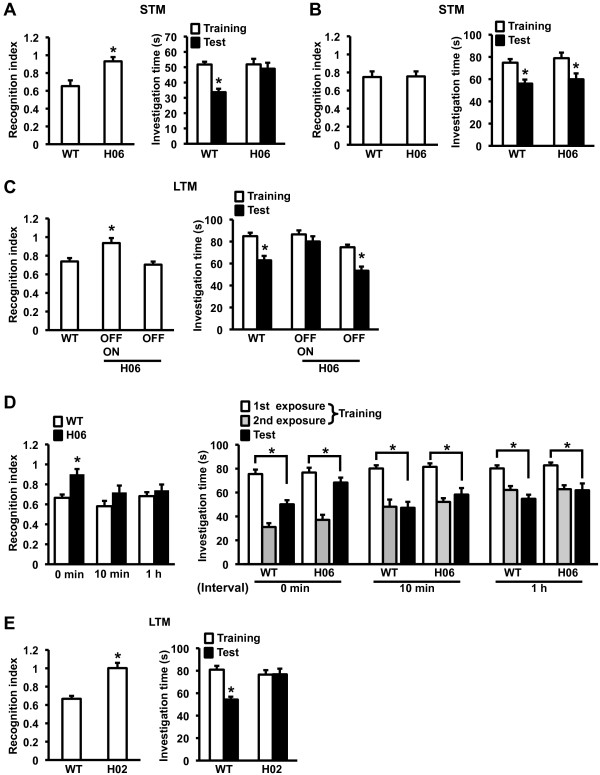
**Impaired social recognition memory in dnRAR mice and its rescue by stronger training**. (A) STM formed by training for 1.5 min (WT, n = 14; H06, n = 15). Recognition index (left panel). *p < 0.05, compared with WT. Investigation time (right panel). *p < 0.05, compared with training. (B) STM formed by training for 3 min (WT, n = 8; H06, n = 10). Recognition index (left panel). *p < 0.05, compared with WT. Investigation time (right panel). *p < 0.05, compared with training. (C) LTM formed by training for 3 min (WT, n = 28; OFF/ON, n = 26; OFF, n = 17). Recognition index (left panel). *p < 0.05, compared with the other groups. Investigation time (right panel). *p < 0.05, compared with training. (D) LTM formed by massed or spaced training (0 min: WT, n = 11; H06, n = 13; 10 min: WT, n = 12; H06, n = 11; 1 h: WT, n = 17; H06, n = 11). Recognition index (left panel). *p < 0.05, compared with the other groups. Investigation time (right panel). *p < 0.05, compared with the first exposure during training. (E) LTM formed by training for 3 min in WT (n = 11) and dnRAR H02 (n = 13) mice. Recognition index (left panel). *p < 0.05. Investigation time (right panel). *p < 0.05, compared with training. Error bars indicate SEM.

To investigate the effects of the impaired RAR/RXR signaling pathway on learning and memory, we performed a hippocampus-dependent social recognition memory task. This task measures the difference in the time taken to investigate a juvenile mouse by comparing between the first (training) and second (test) exposures to the mouse [32,33]. We first examined 2 h-short-term memory (STM; Figure 3A). Mice were exposed to a juvenile mouse for 1.5 min during the training and test sessions, which were performed 2 h apart. We measured the social investigation time of WT and OFF/ON-dnRAR H06 mice with the juvenile mouse during the training and test sessions and assessed the recognition index (i.e., the ratio of the social investigation time at the test relative to the training). One-way ANOVA with genotype (dnRAR and WT) revealed a significant effect of genotype (F_(1,27) _= 12.107, p < 0.05). The *post hoc *Newman-Keuls test revealed that dnRAR H06 mice showed a significantly worse recognition index than the WT mice (p < 0.05). Consistently, comparison of the social investigation time from the training and test sessions indicated that WT mice, but not OFF/ON-dnRAR H06 mice, displayed a significant decrease in social investigation time during the test compared to the training, indicating that WT mice formed STM, while OFF/ON-dnRAR H06 mice did not. These results indicated that OFF/ON-dnRAR H06 mice have impaired STM.

Our finding that the impaired LTP observed in OFF/ON-dnRAR H06 mice was rescued by stronger conditioning stimulation raised the possibility that stronger training could also rescue the impaired STM in OFF/ON-dnRAR H06 mice. To examine this, the mice were tested using a stronger training protocol in which they were exposed to a juvenile mouse for 3 min during the training and test sessions (Figure 3B) [33]. In contrast to the results shown above, one-way ANOVA revealed no significant effect of genotype (F_(1,16) _= 0.006, p > 0.05). Consistently, WT and OFF/ON-dnRAR H06 mice displayed a significant decrease in social investigation time during the test compared with the training, indicating that both groups formed STM. These observations indicated that stronger training rescued the impaired STM observed in dnRAR H06 mice.

We next examined the effects of dnRAR expression on the formation of long-term memory (LTM; Figure 3C). The mice were exposed to a juvenile mouse for 3 min during the training and test sessions, which were performed 24 h apart. In this experiment, OFF-dnRAR H06 mice that were administered Dox throughout their lifetime were also tested as a control group to clarify the effect of dnRAR expression. One-way ANOVA revealed an effect of group (F_(2,68) _= 7.992, p < 0.05). OFF/ON-dnRAR H06 mice displayed a significantly worse recognition index than the WT and OFF-dnRAR H06 mice (p < 0.05), which displayed comparable recognition indices (p > 0.05). Consistently, the control groups (p < 0.05), but not OFF/ON-dnRAR H06 mice (p > 0.05), displayed significant decreases in social investigation time during the test compared with the training, indicating that the control groups formed LTM, while dnRAR H06 mice did not. These observations indicated that dnRAR H06 mice display impaired LTM in a dnRAR expression-dependent manner.

On the basis of our observations in the STM experiment, we examined whether a stronger training protocol rescues the impaired LTM observed in dnRAR H06 mice (Figure 3D). To do this, we used a spaced training protocol because a previous study demonstrated that the impaired LTM observed in mutant mice was rescued by a spaced, but not massed, training protocol [34]. The mice were trained with exposure to a juvenile mice for 3 min twice with an interval of 0 min (massed training), 10 min, or 1 h (spaced training), and 24 h later, they were tested with exposure to the same juvenile mouse for 3 min (test). We assessed the recognition indices, i.e., the ratio of the social investigation time during the test relative to the first exposure during training. Two-way ANOVA with the duration of the interval (0 and 10 min, and 1 h) and genotype revealed an effect of genotype (F_(1,67) _= 11.930, p < 0.05), suggesting that, similar with the result shown in Figure 3C, dnRAR mice displayed impaired LTM. Consistently, the *post hoc *Newman-Keuls test revealed that massed-trained OFF/ON-dnRAR H06 mice displayed a significantly worse recognition index than the other groups (p < 0.05), indicating that massed-trained dnRAR mice exhibited significantly impaired LTM. However, spaced-trained dnRAR mice (10 min or 1 h) displayed a comparable recognition index with the other groups of WT mice (p > 0.05), suggesting that these groups of spaced-trained mutant mice exhibited normal LTM. Consistently, although all groups displayed significant decreases in social investigation time during the test compared with the first exposure during training (p < 0.05), massed-trained OFF/ON-dnRAR H06 mice displayed a significant, but small, decrease in their social investigation time. These observations indicated that the impaired LTM observed in OFF/ON-dnRAR H06 mice was rescued by spaced, but not massed, training with an interval of 10 min-1 h.

We also performed a social recognition test using another transgenic line (dnRAR H02 mice) that displays higher expression levels of dnRAR (Figure 3E). The mice were trained and then tested 24 h later with exposure to a juvenile mouse for 3 min. Consistent with the result for dnRAR H06 mice, one-way ANOVA revealed a significant effect of genotype (F_(1,22) _= 23.568, p < 0.05). OFF/ON-dnRAR-H02 mice showed a significantly worse recognition index than WT mice (p < 0.05). Consistently, WT, but not OFF/ON-dnRAR H02, mice exhibited a significant decrease in social investigation time during the test compared with training (p > 0.05). These observations confirmed that the expression of dnRAR in the forebrain leads to impaired social recognition memory.

Taken together, our observations indicated that dnRAR mice have impaired ST- and LT-social recognition memories. Importantly, these memory impairments are rescued by stronger training.

### Impaired spatial memory in dnRAR mice and its rescue by stronger training

**Figure 4:**
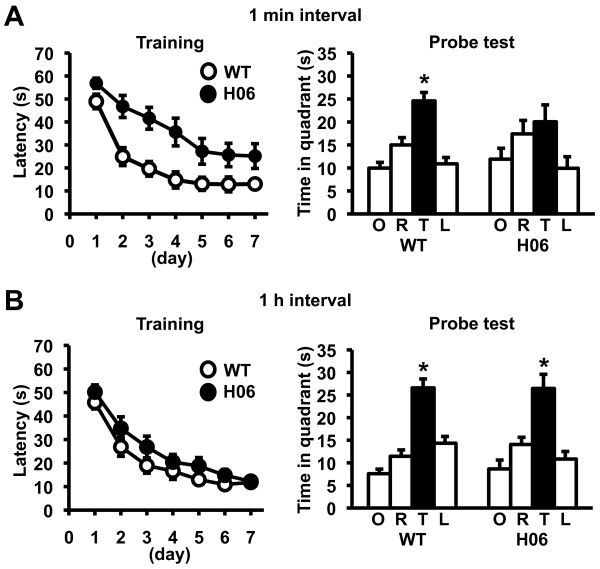
**Impaired spatial memory in dnRAR mice and its rescue by spaced training**. (A) Escape latencies during training with a 1 min interval (left panel; WT, n = 18; H06, n = 12). Data are indicated in blocks of 2 trials. Probe test at day 8 (right panel). *p < 0.05 compared with the other 3 quadrants. (B) Escape latencies during training with a 1 h interval (left panel; WT, n = 15; H06, n = 12). Data are indicated in blocks of 2 trials. Probe test at day 8 (right panel). *p < 0.05 compared with the other 3 quadrants. Error bars indicate SEM.

To examine whether the memory impairment caused by dnRAR expression can be generalized to other hippocampus-dependent memories formed by different sensory, motivational, and performance demands, we performed the Morris water maze test. In this task, the mice formed a spatial memory of the position of a hidden platform in a swimming pool using spatial cues around the pool. The mice were trained with 2 trials at 1 min intervals per day for 7 days (Figure 4A). Two-way repeated ANOVA with genotype (WT and dnRAR) and time (days 1-7) revealed significant effects of genotype and time (genotype, F_(1,26) _= 25.402, p < 0.05; time, F_(6,156) _= 23.833, p < 0.05). The *post hoc *Newman-Keuls test revealed that OFF/ON-dnRAR H06 mice displayed significantly longer escape latencies than WT mice at days 2-7. These results indicated that dnRAR H06 mice displayed a worse performance during training than WT mice. To examine the formation of spatial memory, we performed a probe test at day 8 after training for 7 days. In the probe test, the mice were allowed to swim for 60 s, and we assessed the time spent in each quadrant of the pool (Figure 4A). Planned comparisons using a paired *t *test revealed that WT mice spent significantly more time in the target quadrant (TQ) compared with the other quadrants [TQ vs. opposite (OP), adjacent right (AR), or adjacent left (AL); p < 0.05], while OFF/ON-dnRAR H06 mice did not (p > 0.05), indicating that only WT mice formed spatial memory. These observations indicated that OFF/ON-dnRAR H06 mice have impaired spatial memory.

On the basis of the observations in the social recognition task, it is possible that spaced training could rescue the impaired memory performance observed in dnRAR H06 mice. To examine this, the mice were trained with 2 trials at 1 h intervals per day for 7 days (Figure 4B). In contrast to the previous results, two-way repeated ANOVA revealed a significant effect of time, but not genotype (genotype, F_(1,25) _= 2.872, p > 0.05; time, F_(6,150) _= 46.768, p < 0.05), indicating that both groups of mice displayed a comparable improvement of memory performance as both groups showed decreases in escape latency with training days. Consistently, the results of the probe test revealed that WT and OFF/ON-dnRAR H06 mice spent significantly more time in the TQ compared with the other quadrants, indicating that both groups formed spatial memory. These results indicated that the impaired spatial memory observed in OFF/ON-dnRAR H06 mice was rescued by spaced training. Collectively, these observations indicated that OFF/ON-dnRAR H06 mice have a deficit in spatial memory, but this deficit is rescued by stronger training, and confirmed our findings in the social recognition task.

It is important to note that WT and OFF/ON-dnRAR H06 mice showed normal locomotor activity and anxiety-related behaviors (time spent in the center of field) in the open field test (Additional file 1, Figure S1). Furthermore, OFF/ON-dnRAR H06 mice displayed normal swim speed (Additional file 2, Figure S2) and social investigation time during the training session in social recognition test (Figure 3), compared to WT mice. These observations strongly suggest that memory impairments observed in dnRAR mice does not attribute to abnormal locomotor and/or emotional behaviors such as social and anxiety-related behaviors.

### Pharmacological loss-of-function of hippocampal RARα impairs social recognition memory

**Figure 5:**
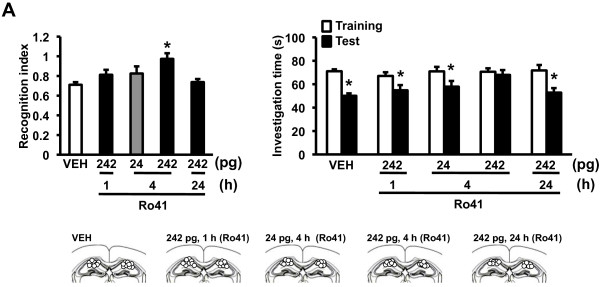
**Impaired social recognition memory by the pharmacological inhibition of hippocampal RARα**. Recognition index (left panel). Effects of micro-infused Ro41-5253 (Ro41; 242 pg/side) into the dorsal hippocampus at 1, 4, or 24 h before training and the effects of micro-infused low-dose Ro41 (24 pg/side) on 24 h LT-social recognition memory (VEH, n = 38; 1 h, n = 12; 4 h-low, n = 11; 4 h, n = 13; 24 h, n = 12). *p < 0.05, compared with the VEH group. Investigation time (right panel). *p < 0.05, compared with training. The lower panel indicates cannula tip placement in mice infused with VEH or Ro41.

Our observations that dnRAR expression in the forebrain led to impairments of hippocampal synaptic transmission and plasticity and the formation of two different types of hippocampus-dependent memories suggest that the RAR/RXR signaling pathway plays a crucial role in hippocampus-dependent memory. To further understand the roles of RARα in the hippocampus, we examined the effects of the pharmacological blockade of RARα in the hippocampus on LT-social recognition memory using a micro-infusion of Ro41-5253 (Ro41), a selective antagonist of RARα, into the dorsal hippocampus. WT mice were trained and tested 24 h later with exposure to a juvenile mouse for 3 min. The mice received a micro-infusion of vehicle (VEH) or Ro41 (242 pg/side) into the dorsal hippocampus at 1, 4, or 24 h, respectively, before training (Figure 5A). One-way ANOVA revealed a significant effect of group (F_(3,53) _= 5.460, p < 0.05). The mice infused with Ro41 at 4 h before training displayed a significantly worse recognition index than the other groups (p < 0.05), which displayed comparable recognition indices (p > 0.05). Consistently, the mice infused with Ro41 at 4 h before training failed to decrease their social investigation time during the test compared with training, while the other groups displayed significant decreases in their social investigation time. These observations indicated that the microinfusion of Ro41 into the hippocampus impairs social recognition memory within a time window that impairs memory performance. We also examined the dose-dependent effects of Ro41 on the impairment of LT-social recognition memory. WT mice received a low-dose microinfusion of Ro41 (24 pg/side) into the dorsal hippocampus at 4 h before training. One-way ANOVA with drug (VEH and the low and high dose groups) revealed a significant effect of group (F_(2,41) _= 8.046, p < 0.05). The mice that received a high dose of Ro41 displayed a significantly worse recognition index than the VEH group (p < 0.05), whereas the mice that received a low dose of Ro41 displayed a comparable recognition index with the mice infused with VEH or a high dose of Ro41 (p > 0.05). Consistently, the mice that were infused with a low dose of Ro41 displayed a significant decrease in social investigation time during the test compared with training, while the mice infused with a high dose of Ro41 did not. These observations indicated that the micro-infusion of Ro41 into the hippocampus impairs social recognition memory in a dose-dependent manner. Collectively, our observations indicated that the blockade of RARα in the hippocampus impairs social recognition memory, suggesting that the RAR/RXR signaling pathway in the hippocampus is crucial for social recognition memory.

## Discussion

In this study, we characterized the roles of the RAR/RXR signaling pathway in the forebrain during learning and memory. To do this, we generated conditional mutant mice in which the expression of dnRAR was induced only in the forebrain of adult mice using a tetracycline-dependent transcription factor expression system. These mutant mice induced dnRAR expression in a Dox-dependent manner and down-regulated RARβ gene expression, a target gene of RAR/RXR, in a dnRAR expression-dependent manner, suggesting that these mutant mice only display a dysfunction of the RAR/RXR signaling pathway in the forebrain when dnRAR is expressed. Similarly with previous findings, these dnRAR mice displayed impaired LTP, especially the maintenance of LTP in the hippocampus; however, this deficit of LTP was rescued by stronger stimulation. Furthermore, we observed an impaired AMPA receptor-mediated synaptic response in dnRAR mice. These findings suggest that the dysfunction of the RAR/RXR signaling pathway impairs synaptic transmission as well as potentiation in the hippocampus. Conversely, dnRAR mice displayed deficits in ST- and LT-social recognition memory without an effect on immediate memory (see Figure 3D). Interestingly, these impairments of STM and LTM in the dnRAR mice were rescued by stronger or spaced training, respectively. Consistently, dnRAR mice displayed normal spatial learning and memory in the Morris water maze when they were spaced trained, but not when they were massed trained. Furthermore, pharmacological inhibition of RARα in the hippocampus impaired social recognition memory. These behavioral observations suggest that the RAR/RXR signaling pathway plays critical roles in hippocampus-dependent memory. Taken together, our observations strongly suggest that the RAR/RXR signaling pathway is required for synaptic transmission and plasticity and learning and memory in the forebrain, especially the hippocampus.

Memory consolidation is a process that generates LTM [35-37]. Previous studies have shown that the inhibition of gene expression blocks the formation of LTM without affecting STM [38-40]. Indeed, the activation of CREB-mediated transcription plays an essential role in memory consolidation and LTP [33,39]. In this study, we showed that dysfunction of the RAR/RXR signaling pathway impairs STM and LTM. These observations suggest that RAR/RXR play distinct roles from CREB in hippocampus-dependent memory; it is unlikely that RAR/RXR greatly contribute to memory consolidation via learning-induced RAR/RXR-mediated transcription, as CREB does. Indeed, our finding that loss of RAR/RXR function impairs not only LTP but also the AMPA receptor-mediated synaptic response raises the possibility that RAR/RXR contribute to leaning/memory via basal synaptic transmission as well as long-term plasticity.

Interestingly, we observed that RAR/RXR dysfunction impaired the hippocampal LTP induced by a single conditioning stimulus and hippocampus-dependent social and spatial memories. However, stronger stimulation (four conditioning stimuli) and stronger or spaced training rescued these impairments in LTP and memories, respectively. The similarity of the observations at the cellular and behavioral levels strongly suggests the importance of the RAR/RXR signaling pathway in the hippocampus for both plasticity and learning and memory. Furthermore, this importance is supported by our observations that the pharmacological inhibition of RARα function in the hippocampus blocked the formation of social recognition memories. Further studies are required to examine the correlation between synaptic plasticity and learning/memory ability in the hippocampus of dnRAR mice, and the roles of RAR-mediated long-term plasticity in memory performance.

Recent reports have shown that RARα is present in the dendrites located in the hippocampus and induces the translation of GluA1 in a RA-dependent manner, resulting in the increased membrane insertion of GluA1 [41,42]. Interestingly, this regulation of GluA1 by RARα is independent of transcriptional regulation [42,43]. In the absence of RA, cytoplasmic RARα directly binds with GluA1 mRNA localized in dendrites via its C-terminal F-domain and represses its translation [44]. In the presence of RA, RA binding to RARα releases the GluA1 mRNA that is trapped by RARα, leading to the translational activation of GluA1 [44]. The dominant-negative mutant of RARα used in this study lacks the C-terminus, including the F-domain that is required for its interaction with GluA1 mRNA [26]. Therefore, it is possible that the dnRAR used in this study not only inhibits RAR/RXR-mediated transcription but also affects the translational regulation of GluA1. dnRAR might block the RA-dependent release of GluA1 mRNA in dendrites by trapping RA and inhibiting the translational activation of GluA1 mRNA, resulting in the down-regulation of AMPA-mediated synaptic transmission.

The findings in this and other studies showed that the dysfunction of RAR/RXR leads to impairments of memory and LTP in the hippocampus [18-20]. Conversely, previous studies have suggested that the age-related down-regulation of RAR/RXR-mediated gene expression is implicated in the cognitive decline of aged mice [16,17]. Importantly, these studies demonstrated that the administration of RA alleviates the down-regulated expression of RA-target genes in the hippocampus, age-related deficits in hippocampal LTP, and memory performance. Furthermore, the administration of RA prevents the deposition of Aβ and rescues memory deficits in a transgenic mouse model of Alzheimer' disease [45]. Taken together, these observations raise the possibility that the RAR/RXR signaling pathway positively regulates memory performance and, moreover, that the activation of this signaling pathway improves learning and memory. Further studies are required to examine the effects of gain of RAR/RXR function on learning and memory by the genetic enhancement of RAR/RXR.

A previous genetic study showed that the deletion of the RARβ or RARβ/RXRγ genes leads to disrupted LTP and LTD in the CA1 region of the hippocampus without affecting basal synaptic transmission [18]. In contrast, our results indicated that dysfunction of the RAR/RXR signaling pathway by the expression of dnRAR impairs the maintenance, but not induction, of CA1-LTP and the AMPA receptor-mediated synaptic response. The differences between these two studies may reflect distinct roles of RAR/RXR subtypes in synaptic transmission and potentiation in the hippocampus [6,46]. Additionally, there is a possibility that the protocols used to induce LTP contributed to the different findings of these two studies.

In summary, we found that dysfunction of the RAR/RXR signaling pathway leads to the down-regulation of RARβ expression and impairment of AMPA-mediated synaptic transmission and LTP in the hippocampus and hippocampus-dependent social recognition and spatial memories. More interestingly, stronger conditioning stimulation and training rescued these impairments of LTP and memory performance, respectively. Furthermore, we showed that blocking the function of RARα in the hippocampus disrupted social recognition memory. These observations strongly suggested that the RAR/RXR signaling pathway in the hippocampus plays critical roles in synaptic plasticity and memory performance.

## Materials and methods

### Mice

Mice were housed in cages of 5 or 6, maintained on a 12 h light/dark schedule, and allowed *ad libitum *access to food and water in their home cages. All of the experiments were conducted during the light phase of the cycle in an illuminated testing room according to the *Guide for the Care and Use of Laboratory Animals, Japan Neuroscience Society and Tokyo University of Agriculture*. All of the experiments were conducted blind to the treatment condition of the mouse. Animal behavior was recorded using a video camera.

### Plasmid construction

Generation of the dominant-negative RARα mutant (dnRAR) lacking the C-terminal region of RARα (aa 403-462) was performed as described previously [26]. To generate dnRAR cDNA, 2 separate fragments of the dnRAR cDNA (nucleotides 1-438 and 433-1206) were amplified by reverse transcription-polymerase chain reaction (RT-PCR) using mouse brain cDNA as a template with the following primers: 1/438 primers (sense, gggggatccagatctatggccagcaatagcagttc; antisense, ggggaattcctgcagccggcagtactggc); 433/1209 primers (sense, gggggatccctgcagaaatgtttcgacgt; antisense, ggggaattcacgcgtaagctttcagatctccatcttcaatg; the termination codon to generate dnRAR is underlined). The resulting RT-PCR fragments were subcloned into the *Bam*HI-*Eco*RI sites of pBluescript II (SK+) (Agilent Technologies, CA, USA), generating pBS-dnRAR. The *Bgl*II-*Hind*III fragment encoding dnRAR from pBS-dnRAR was ligated with the *Bgl*II-*Hind*III fragment from pTRE-dnBMAL1 [27], generating pTRE-dnRAR.

### Generation of transgenic mice

pTRE-dnRAR was digested with *Mlu*I, and transgenic mice were generated by injecting the purified insert into the pronuclei of C57BL/6N zygotes. TRE-dnRAR founders were crossed with C57BL/6N mice (Charls River Japan, Kanagawa, Japan). These founders were crossed with mice (CaMKII-tTA) expressing the tTA transgene under the control of the αCaMKII promoter [24], generating double-transgenic mice (dnRAR TgM). Genotyping was performed by Southern blot analysis using specific probes derived from the 0.5 kbp *Not*I-*Nde*I fragment containing the TRE-promoter region from pcisTRE [27] and the 0.6 kbp *Eco*RI-*Hind*III fragment containing the tTA-coding region from pcDNA3-TetR-KRAB [27].

### Administration of doxycycline

TRE promoter-dependent transgene expression was regulated using the animal's drinking water containing 1 μg/mL doxycycline (Dox) (Sigma, MO, USA) dissolved in 5% sucrose to mask the bitter taste of Dox.

### Biochemical experiments

Northern blot analysis was performed as described previously [39,47]. Total RNA was prepared as described previously [39,47]. To generate a specific probe for TRE promoter-dependent transcripts, the 0.3 kbp *Hind*III-*Xba*I fragment containing the 3'-UTR from pTRE-dnRAR was used. The filters were hybridized with a specific probe for the 3'-UTR and then re-hybridized with GAPDH cDNA as an internal control. qRT-PCR was performed as described previously [47]. qRT-PCR was performed on an ABI PRISM 7000 using the Power SYBR Green PCR Master Mix (Applied Biosystems, CA, USA) according to the manufacturer's protocol. The reaction was first incubated at 50°C for 2 min, then at 95°C for 10 min, followed by 40 cycles of 95°C for 15 s and 60°C for 1 min. All measurements were performed in triplicate. The levels of GAPDH mRNA were used to normalize the relative expression levels of target mRNA. The PCR primers used were as follows (5' to 3'): RARβ forward, tcctggatcaatgccacctc; RARβ reverse, acacgctggactgtgctct; GAPDH forward, atggccttccgtgttcctac; and GAPDH reverse, gcctgcttcaccaccttctt. Western blot analysis using a rabbit polyclonal anti-HA antibody (1:1000; Santa Cruz Biotechnology, CA, USA) and a mouse monoclonal anti-α-tubulin antibody (1:1000; Santa Cruz Biotechnology, CA, USA) was performed as described previously [48,49]. Equal protein loading was confirmed by reprobing with the anti-α-tubulin antibody. The protein bands were detected using the ImmunoStar LD system (Wako, Osaka, Japan) according to the manufacturer's protocol, and the chemiluminescence signals were analyzed with the ChemiDoc XRS detection system and QuantityOne software (Bio-Rad Laboratories, CA, USA).

### Behavioral experiments

Before the commencement of the behavioral experiments, the mice were individually handled for 2 min each day for 1 week. Individual mice were used for all experiments.

### Social recognition test

The social recognition test was performed as described previously [32,33]. Adult mice were placed into individual plastic cages in an experimental room under dim light. The cages were identical to those in which mice were normally housed (plastic, 30 × 17 × 12 cm). After a period of 60 min, a juvenile mouse was placed into a cage with a subject for a training trial that lasted for 1.5 min (see Figure 3A) or 3 min (see Figures 3B, C, E, 5A). The duration of social investigation behavior exhibited by the adult mouse was determined using a hand-held stopwatch. Social investigation was measured as described previously [50]. Memory was reassessed at 2 h or 24 h later by recording the length of the investigation time exhibited by the subject to the same juvenile (test). To evaluate the differences in the ability to form social memory between the groups of mice, we calculated a recognition index, i.e., the ratio of the duration of the second and first investigation times. When massed or spaced training was performed, the subjects were trained with 2 trials lasting 3 min at intervals of 0 min (massed-training) and 10 min, 1 h (spaced training) on the first trial day. Memory was reassessed at 24 h after the training as described above. The recognition index was calculated as the ratio of the social investigation time during the test relative to the first exposure during training.

### Morris water maze test

The Morris water maze test was performed as described previously [33,40,51]. The mice were trained with 2 trials at intervals of 1 min (see Figure 4A) or 1 h (see Figure 4B) per day for 7 days. The mice were tested at approximately the same time everyday. In the probe test at 24 h after 7 d of training, the platform was removed, and the mice were allowed to swim for 60 s. We measured the time that the mice spent in each quadrant.

### Surgery for drug microinfusion

Surgery was performed as described previously [51-54]. Under Nembutal anesthesia and using standard stereotaxic procedures, stainless steel guide cannulae (22 gauge) were implanted into the dorsal hippocampus (-1.8 mm, ± 1.8 mm, -1.9 mm). Ro41-5253 (Ro41) (Enzo Life Sciences, NY, USA), an RARα-selective antagonist, was dissolved in dimethyl sulfoxide (DMSO) (Wako, Osaka, Japan), and then dissolved in vehicle solution (VEH) [artificial CSF (ACSF)] with a final DMSO concentration of 0.01%. Micro-infusions into each brain region (0.5 μL) were made at a rate of 0.25 μL/min. The injection cannula was left in place for 2 min after the infusion.

### Electrophysiology

Electrophysiology was performed as described previously [30,55]. All experiments were performed to compare dnRAR mice with WT mice in a blind fashion using littermates. Three- to 5-month-old dnRAR and WT mice were decapitated under deep halothane anesthesia, and both hippocampi were removed. Hippocampal slices (400-μm thick) were cut with a Vibratome tissue slicer and placed in a humidified interface-type chamber for at least 1 h. A single slice was then transferred to the recording chamber, which was maintained at 25°C, and submerged beneath a continuously perfusing medium (119 mM NaCl, 2.5 mM KCl, 1.3 mM MgSO_4_, 2.5 mM CaCl_2_, 1.0 mM NaH_2_PO_4_, 26.2 mM NaHCO_3_, and 11 mM glucose) that had been saturated with 95% O_2 _and 5% CO_2_. All perfusing solutions contained picrotoxin (100 μM) to block gamma-aminobutyric acid A receptor (GABA-A-R)-mediated inhibitory synaptic responses. Field potential recordings were made using a glass electrode filled with 3 M NaCl and placed in the stratum radiatum of the hippocampal CA1 region.

To evoke synaptic responses, a bipolar stimulating tungsten electrode was placed in the stratum radiatum, and Schaffer collateral/commissural fibers were stimulated at 0.1 Hz (test pulses). An Axopatch 200B amplifier (Molecular Devices, Sunnyvale, USA) was used, and the signal was filtered at 1 kHz and digitized at 10 kHz, and stored on a personal computer. The stimulus strength was adjusted so that it gave rise to AMPA receptor-mediated EPSPs with a slope value between 0.10 and 0.15 mV/ms. For the analysis of EPSPs, we measured their early rising phase to avoid contamination from voltage-dependent components as much as possible. Each data point represents the averaged slope value for 1 min that was normalized to the baseline slope value. For LTP recording, LTP was induced using 1 or 4 high-frequency stimulations (one 100 Hz, 1 s train or four 100 Hz, 1 s trains at 5 min intervals, respectively). For STP recording, STP was induced using a single high-frequency stimulation (one 100 Hz, 100 ms train). To record the input-output relationships, D-2-amino-5-phosphonovaleric acid (D-APV, 25 μM) was present to block N-methyl-D-aspartate receptor (NMDA-R)-mediated synaptic responses. A low concentration of 6-cyano-7-nitro-quinoxaline-2,3-dione (CNQX, 1 μM) was also present to partially block alpha-amino-3-hydroxy-5-methyl-4-isoxazole propionate receptor (AMPA-R)-mediated synaptic responses because the fiber volleys were usually much smaller than the EPSPs. This enables more accurate measurements of the input-output relationships, since the presence of low concentrations of CNQX reduces the nonlinear summation of field EPSPs when strong stimulus strengths are used. For the measurement of PPF, afferent fibers were stimulated twice at intervals of 50, 100, 200, and 300 ms in the presence of D-APV (25 μM). For PTP recordings, PTP was induced using a single high-frequency stimulation (one 100 Hz, 1 s train) in the presence of D-APV (50 μM). Data were collected using Clampex 5.0 and analyzed with pClamp 9.0 software. Picrotoxin and D-APV were purchased from Sigma (MO, USA) and CNQX was purchased from Tocris Cookson (Avonmouth, UK).

### Data analysis

Data were analyzed with ANOVA. One-way ANOVA analysis followed by the *post hoc *Newman-Keuls comparison was used to analyze the effects of genotype, time, and drug. Two-way ANOVA analysis followed by the *post hoc *Newman-Keuls comparison was used to analyze the effects of genotype, time, and duration of the interval. All values in the text and figure legends represent the mean ± SEM. A paired *t *test was used to analyze the differences in the social investigation times within each group between training and test in the social recognition test, and to analyze the differences of the time spent in the TQ compared with the other quadrants in the Morris water maze test. Student's *t *test (two-tailed, unpaired) was used to determine whether there was a significant difference in the means between two sets of data in the electrophysiological experiments.

## List of abbreviations

tTA: tetracycline-controlled transactivator; αCaMKII: α-calcium/calmodulin-dependent protein kinase II.

## Competing interests

The authors declare that they have no competing interests.

## Authors' contributions

SKida is responsible for the hypothesis development and overall design of the research and experiment, and supervised the experimental analyses. SKida and MNomoto co-wrote the manuscript. MNomoto performed biochemical and electro-physiological analyses. YTakeda, HEnomoto, and TChoi performed behavioral analyses. SUchida constructed a transgene and generated transgenic mice expressing dnRAR. KMitsuda and KSaito performed treatment of doxycycline. AM. Watabe, SKobayashi, SMasushige, and TManabe supervised experimental analyses. All authors read and approved this manuscript.

## Supplementary Material

Additional file 1 **Figure S1. Locomotion and anxiety-related behaviors of dnRAR mice in the open field test**. The open field test was performed as described previously [47]. Mice were placed into the center of a square open field chamber (40 cm long × 40 cm wide × 40 cm high) that was surrounded by white acrylic walls. The total length of the path mice traveled (locomotor activity) and the time they spent in a center square (24 cm × 24 cm;% center) were measured over the course of 5 min using an automatic monitoring system (Neuroscience Inc., Tokyo, Japan). (A) The total path length for 5 min. (B) The percent of time spent in the center for 5 min. WT (n = 8) and OFF/ON-dnRAR H06 mice (n = 8) showed comparable total path and percentage of time spent in the center of the field (one-way ANOVA; locomotor activity, F_(1,14) _= 0.035, P > 0.05;% center, F_(1,14) _= 0.597, P > 0.05). These results suggested that OFF/ON-dnRAR H06 mice display normal locomotor activity and anxiety-related behaviors. Error bars are SEM. Click here for file

Additional file 2 **Figure S2. Comparison of swim speed between WT and dnRAR mice**. Mice were allowed to swim in the pool used in Morris water maze test. The total length of the path for 1 min (total path) using an automatic monitoring system (Neuroscience Inc., Tokyo, Japan) and then swim speed was calculated. WT (n = 11) and OFF/ON dnRAR H06 (n = 15) mice showed comparable swim speed (one-way ANOVA; F_(1,24) _= 0.023, P > 0.05). Error bars are SEM. Click here for file

## References

[B1] Mangelsdorf DJ Vitamin A receptors Nutr Rev 1994 52 S32 44 8202281 10.1111/j.1753-4887.1994.tb01385.x

[B2] Sucov HM Evans RM Retinoic acid and retinoic acid receptors in development Mol Neurobiol 1995 10 169 184 10.1007/BF02740674 7576306

[B3] Napoli JL Retinoic acid biosynthesis and metabolism FASEB J 1996 10 993 1001 8801182 10.1096/fasebj.10.9.8801182

[B4] Chambon P A decade of molecular biology of retinoic acid receptors FASEB J 1996 10 940 954 8801176

[B5] Evans RM The steroid and thyroid hormone receptor superfamily Science 1988 240 889 895 10.1126/science.3283939 3283939 PMC6159881

[B6] Mangelsdorf DJ Thummel C Beato M Herrlich P Schütz G Umesono K Blumberg B Kastner P Mark M Chambon P Evans RM The nuclear receptor superfamily: the second decade Cell 1995 6 835 839 10.1016/0092-8674(95)90199-x PMC6159888 8521507

[B7] McCaffery P Zhang J Crandall JE Retinoic acid signaling and function in the adult hippocampus J Neurobiol 2006 7 780 791 10.1002/neu.20237 16688774

[B8] Gofflot F Chartoire N Vasseur L Heikkinen S Dembele D Le Merrer J Auwerx J Systematic gene expression mapping clusters nuclear receptors according to their function in the brain Cell 2007 2 405 418 10.1016/j.cell.2007.09.012 17956739

[B9] Goodman AB Three independent lines of evidence suggest retinoids as causal to schizophrenia Proc Natl Acad Sci USA 1998 13 7240 7244 10.1073/pnas.95.13.7240 PMC33865 9636132

[B10] Goodman AB Pardee AB Evidence for defective retinoid transport and function in late onset Alzheimer's disease Proc Natl Acad Sci USA 2003 5 2901 2905 10.1073/pnas.0437937100 PMC151438 12604774

[B11] Corcoran JP So PL Maden M Disruption of the retinoid signalling pathway causes a deposition of amyloid beta in the adult rat brain Eur J Neurosci 2004 4 896 902 10.1111/j.1460-9568.2004.03563.x 15305858

[B12] Goodman AB Retinoid receptors, transporters, and metabolizers as therapeutic targets in late onset Alzheimer disease J Cell Physiol 2006 3 598 603 10.1002/jcp.20784 17001693

[B13] Palha JA Goodman AB Thyroid hormones and retinoids: a possible link between genes and environment in schizophrenia Brain Res Rev 2006 1 61 71 10.1016/j.brainresrev.2005.10.001 16325258

[B14] Husson M Enderlin V Delacourte A Ghenimi N Alfos S Pallet V Higueret P Retinoic acid normalizes nuclear receptor mediated hypo-expression of proteins involved in beta-amyloid deposits in the cerebral cortex of vitamin A deprived rats Neurobiol Dis 2006 1 1 10 10.1016/j.nbd.2006.01.008 16531051

[B15] Bremner JD McCaffery P The neurobiology of retinoic acid in affective disorders Prog Neuropsychopharmacol Biol Psychiatry 2008 2 315 331 10.1016/j.pnpbp.2007.07.001 PMC2704911 17707566

[B16] Etchamendy N Enderlin V Marighetto A Vouimba RM Pallet V Jaffard R Higueret P Alleviation of a selective age-related relational memory deficit in mice by pharmacologically induced normalization of brain retinoid signaling J Neurosci 2001 16 6423 6429 10.1523/JNEUROSCI.21-16-06423.2001 PMC6763177 11487666

[B17] Mingaud F Mormede C Etchamendy N Mons N Niedergang B Wietrzych M Pallet V Jaffard R Krezel W Higueret P Marighetto A Retinoid hyposignaling contributes to aging-related decline in hippocampal function in short-term/working memory organization and long-term declarative memory encoding in mice J Neurosci 2008 1 279 291 10.1523/JNEUROSCI.4065-07.2008 PMC6671152 18171945

[B18] Chiang MY Misner D Kempermann G Schikorski T Giguère V Sucov HM Gage FH Stevens CF Evans RM An essential role for retinoid receptors RARbeta and RXRgamma in long-term potentiation and depression Neuron 1998 6 1353 1361 10.1016/s0896-6273(00)80654-6 9883728

[B19] Misner DL Jacobs S Shimizu Y de Urquiza AM Solomin L Perlmann T De Luca LM Stevens CF Evans RM Vitamin A deprivation results in reversible loss of hippocampal long-term synaptic plasticity Proc Natl Acad Sci USA 2001 20 11714 11719 10.1073/pnas.191369798 PMC58795 11553775

[B20] Etchamendy N Enderlin V Marighetto A Pallet V Higueret P Jaffard R Vitamin A deficiency and relational memory deficit in adult mice: relationships with changes in brain retinoid signalling Behav Brain Res 2003 1-2 37 49 10.1016/s0166-4328(03)00099-8 14529804

[B21] Krezel W Ghyselinck N Samad TA Dupé V Kastner P Borrelli E Chambon P Impaired locomotion and dopamine signaling in retinoid receptor mutant mice Science 1998 5352 863 867 10.1126/science.279.5352.863 9452386

[B22] Gossen M Bujard H Tight control of gene expression in mammalian cells by tetracycline-responsive promoters Proc Natl Acad Sci USA 1992 12 5547 5551 10.1073/pnas.89.12.5547 PMC49329 1319065

[B23] Furth PA St Onge L Böger H Gruss P Gossen M Kistner A Bujard H Hennighausen L Temporal control of gene expression in transgenic mice by a tetracycline-responsive promoter Proc Natl Acad Sci USA 1994 20 9302 9306 10.1073/pnas.91.20.9302 PMC44800 7937760

[B24] Mayford M Bach ME Huang YY Wang L Hawkins RD Kandel ER Control of memory formation through regulated expression of a CaMKII transgene Science 1996 5293 1678 1683 10.1126/science.274.5293.1678 8939850

[B25] Gu XL Long CX Sun L Xie C Lin X Cai H Astrocytic expression of Parkinson's disease-related A53T alpha-synuclein causes neurodegeneration in mice Mol Brain 2010 3 12 10.1186/1756-6606-3-12 20409326 PMC2873589

[B26] Damm K Heyman RA Umesono K Evans RM Functional inhibition of retinoic acid response by dominant negative retinoic acid receptor mutants Proc Natl Acad Sci USA 1993 7 2989 2993 10.1073/pnas.90.7.2989 PMC46222 8096643

[B27] Uchida S Sakai S Furuichi T Hosoda H Toyota K Ishii T Kitamoto A Sekine M Koike K Masushige S Murphy G Silva AJ Kida S Tight regulation of transgene expression by tetracycline-dependent activator and repressor in brain Genes Brain Behav 2006 1 96 106 10.1111/j.1601-183X.2005.00139.x 16436193

[B28] Perlmann T Rangarajan PN Umesono K Evans RM Determinants for selective RAR and TR recognition of direct repeat HREs Genes Dev 1993 7B 1411 1422 10.1101/gad.7.7b.1411 8392478

[B29] Shimuta M Yoshikawa M Fukaya M Watanabe M Takeshima H Manabe T Postsynaptic modulation of AMPA receptor-mediated synaptic responses and LTP by the type 3 ryanodine receptor Mol Cell Neurosci 2001 5 921 930 10.1006/mcne.2001.0981 11358488

[B30] Niisato K Fujikawa A Komai S Shintani T Watanabe E Sakaguchi G Katsuura G Manabe T Noda M Age-dependent enhancement of hippocampal long-term potentiation and impairment of spatial learning through the Rho-associated kinase pathway in protein tyrosine phosphatase receptor type Z-deficient mice J Neurosci 2005 5 1081 1088 10.1523/JNEUROSCI.2565.04.2005 PMC6725950 15689543

[B31] Zucker RS Regehr WG Short-term synaptic plasticity Annu Rev Physiol 2002 64 355 405 10.1146/annurev.physiol.64.092501.114547 11826273

[B32] Fukushima H Maeda R Suzuki R Suzuki A Nomoto M Toyoda H Wu LJ Xu H Zhao MG Ueda K Kitamoto A Mamiya N Yoshida T Homma S Masushige S Zhuo M Kida S Upregulation of calcium/calmodulin-dependent protein kinase IV improves memory formation and rescues memory loss with aging J Neurosci 2008 40 9910 9919 10.1523/JNEUROSCI.2625-08.2008 PMC6671266 18829949

[B33] Suzuki A Fukushima H Mukawa T Toyoda H Wu LJ Zhao MG Xu H Shang Y Endoh K Iwamoto T Mamiya N Okano E Hasegawa S Mercaldo V Zhang Y Maeda R Ohta M Josselyn SA Zhuo M Kida S Upregulation of CREB-mediated transcription enhances both short- and long-term memory J Neurosci 2011 24 8786 802 10.1523/JNEUROSCI.3257-10.2011 PMC6622960 21677163

[B34] Kogan JH Frankland PW Blendy JA Coblentz J Marowitz Z Schütz G Silva AJ Spaced training induces normal long-term memory in CREB mutant mice Curr Biol 1997 1 1 11 10.1016/S1367-5931(97)80100-7 8999994

[B35] Squire LR Alvarez P Retrograde amnesia and memory consolidation: a neurobiological perspective Curr Opin Neurobiol 1995 5 169 177 10.1016/0959-4388(95)80023-9 7620304

[B36] Dudai Y Consolidation, fragility and the road to the engram Neuron 1996 17 367 370 10.1016/S0896-6273(00)80168-3 8816699

[B37] McGaugh JL Memory-a century of consolidation Science 2000 287 248 251 10.1126/science.287.5451.248 10634773

[B38] Abel T Nguyen PV Barad M Deuel TA Kandel ER Bourtchouladze R Genetic demonstration of a role for PKA in the late phase of LTP and in hippocampus-based long-term memory Cell 1997 5 615 626 10.1016/s0092-8674(00)81904-2 9054501

[B39] Kida S Josselyn SA Peña de Ortiz S Kogan JH Chevere I Masushige S Silva AJ CREB required for the stability of new and reactivated fear memories Nat Neurosci 2002 4 348 355 10.1038/nn819 11889468

[B40] Suzuki A Josselyn SA Frankland PW Masushige S Silva AJ Kida S Memory reconsolidation and extinction have distinct temporal and biochemical signatures J Neurosci 2004 20 4787 4795 10.1523/JNEUROSCI.5491-03.2004 PMC6729467 15152039

[B41] Chen N Napoli JL All-trans-retinoic acid stimulates translation and induces spine formation in hippocampal neurons through a membrane-associated RARalpha FASEB J 2008 1 236 245 10.1096/fj.07-8739com 17712061

[B42] Aoto J Nam CI Poon MM Ting P Chen L Synaptic signaling by all-trans retinoic acid in homeostatic synaptic plasticity Neuron 2008 2 308 320 10.1016/j.neuron.2008.08.012 PMC2634746 18957222

[B43] Maghsoodi B Poon MM Nam CI Aoto J Ting P Chen L Retinoic acid regulates RARalpha-mediated control of translation in dendritic RNA granules during homeostatic synaptic plasticity Proc Natl Acad Sci USA 2008 41 16015 16020 10.1073/pnas.0804801105 PMC2572971 18840692

[B44] Poon MM Chen L Retinoic acid-gated sequence-specific translational control by RARalpha Proc Natl Acad Sci USA 2008 51 20303 20308 10.1073/pnas.0807740105 PMC2629326 19073915

[B45] Ding Y Qiao A Wang Z Goodwin JS Lee ES Block ML Allsbrook M McDonald MP Fan GH Retinoic acid attenuates beta-amyloid deposition and rescues memory deficits in an Alzheimer's disease transgenic mouse model J Neurosci 2008 45 11622 11634 10.1523/JNEUROSCI.3153-08.2008 PMC3844785 18987198

[B46] Nagpal S Saunders M Kastner P Durand B Nakshatri H Chambon P Promoter context- and response element-dependent specificity of the transcriptional activation and modulating functions of retinoic acid receptors Cell 1992 6 1007 1019 10.1016/0092-8674(92)90250-g 1326406

[B47] Hasegawa S Furuichi T Yoshida T Endoh K Kato K Sado M Maeda R Kitamoto A Miyao T Suzuki R Homma S Masushige S Kajii Y Kida S Transgenic up-regulation of alpha-CaMKII in forebrain leads to increased anxiety-like behaviors and aggression Mol Brain 2009 2 6 10.1186/1756-6606-2-6 19257910 PMC2660323

[B48] Hosoda H Motohashi J Kato H Masushige S Kida S A BMAL1 mutant with arginine 91 substituted with alanine acts as a dominant negative inhibitor Gene 2004 2 235 241 10.1016/j.gene.2004.05.022 15315827

[B49] Hosoda H Kato K Asano H Ito M Kato H Iwamoto T Suzuki A Masushige S Kida S CBP/p300 is a cell type-specific modulator of CLOCK/BMAL1-mediated transcription Mol Brain 2009 2 34 10.1186/1756-6606-2-34 19922678 PMC2785803

[B50] Thor DH Wainwright KL Holloway WR Persistence of attention to a novel conspecific: some developmental variables in laboratory rats Dev Psychobiol 1982 1 1 8 10.1002/dev.420150102 7054012

[B51] Kim R Moki R Kida S Molecular mechanisms for the destabilization and restabilization of reactivated spatial memory in the Morris water maze Mol Brain 2011 4 9 10.1186/1756-6606-4-9 21314917 PMC3045328

[B52] Suzuki A Mukawa T Tsukagoshi A Frankland PW Kida S Activation of LVGCCs and CB1 receptors required for destabilization of reactivated contextual fear memories Learn Mem 2008 6 426 433 10.1101/lm.888808 PMC2414253 18511694

[B53] Mamiya N Fukushima H Suzuki A Matsuyama Z Homma S Frankland PW Kida S Brain region-specific gene expression activation required for reconsolidation and extinction of contextual fear memory J Neurosci 2009 2 402 413 10.1523/JNEUROSCI.4639-08.2009 PMC6664934 19144840

[B54] Zhang Y Fukushima H Kida S Induction and requirement of gene expression in the anterior cingulate cortex and medial prefrontal cortex for the consolidation of inhibitory avoidance memory Mol Brain 2011 4 4 10.1186/1756-6606-4-4 21244716 PMC3035037

[B55] Manabe T Togashi H Uchida N Suzuki SC Hayakawa Y Yamamoto M Yoda H Miyakawa T Takeichi M Chisaka O Loss of cadherin-11 adhesion receptor enhances plastic changes in hippocampal synapses and modifies behavioral responses Mol Cell Neurosci 2000 6 534 546 10.1006/mcne.2000.0849 10860580

