# Supplementary figures and images for: Dysfunction of the RAR/RXR signaling pathway in the forebrain impairs hippocampal memory and synaptic plasticity
Source: Mol Brain. 2012 Feb 8;5:8. doi: 10.1186/1756-6606-5-8 (PMC3298701; doi:10.1186/1756-6606-5-8)

**A**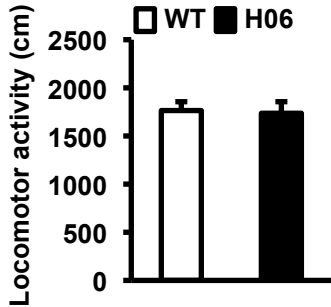**B**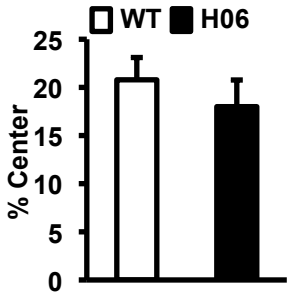

Supplement: Additional file 1 — Figure S1. Locomotion and anxiety-related behaviors of dnRAR mice in the open field test. The open field test was performed as described previously [47]. Mice were placed into the center of a square open field chamber (40 cm long × 40 cm wide × 40 cm high) that was surrounded by white acrylic walls. The total length of the path mice traveled (locomotor activity) and the time they spent in a center square (24 cm × 24 cm;% center) were measured over the course of 5 min using an automatic monitoring system (Neuroscience Inc., Tokyo, Japan). (A) The total path length for 5 min. (B) The percent of time spent in the center for 5 min. WT (n = 8) and OFF/ON-dnRAR H06 mice (n = 8) showed comparable total path and percentage of time spent in the center of the field (one-way ANOVA; locomotor activity, F(1,14) = 0.035, P > 0.05;% center, F(1,14) = 0.597, P > 0.05). These results suggested that OFF/ON-dnRAR H06 mice display normal locomotor activity and anxiety-related behaviors. Error bars are SEM. [file 1756-6606-5-8-S1.PDF]

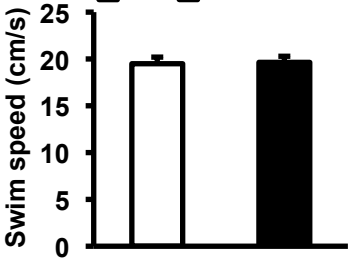

Supplement: Additional file 2 — Figure S2. Comparison of swim speed between WT and dnRAR mice. Mice were allowed to swim in the pool used in Morris water maze test. The total length of the path for 1 min (total path) using an automatic monitoring system (Neuroscience Inc., Tokyo, Japan) and then swim speed was calculated. WT (n = 11) and OFF/ON dnRAR H06 (n = 15) mice showed comparable swim speed (one-way ANOVA; F(1,24) = 0.023, P > 0.05). Error bars are SEM. [file 1756-6606-5-8-S2.PDF]
